# Challenges and Opportunities in China’s Health Aid to Africa: Findings from Qualitative Interviews in Tanzania and Malawi

**DOI:** 10.1186/s12992-020-00577-0

**Published:** 2020-07-29

**Authors:** Garrison Daly, Joan Kaufman, Shuang Lin, Liangmin Gao, Melissa Reyes, Sarah Matemu, Wafaa El-Sadr

**Affiliations:** 1 Mailman School of Public Health, Columbia University, 722 West 168th Street, New York, NY 10032 USA; 2 Schwarzman Scholars, 641 Lexington Avenue, 23rd Floor, New York, NY 10022 USA; 3 Global Health and Social Medicine, Harvard Medical School, 641 Huntington Avenue, Boston, MA 02115 USA; 4 School of International and Public Affairs, Columbia University, 420 West 118th Street, New York, NY 10027 USA; 5 School of Social Sciences, Tsinghua University, Beijing, 100084 China; 6 ICAP in Tanzania, Dar es Salaam, Tanzania; 7 CAP at Columbia University, 722 West 168th Street, New York, NY 10032 USA

**Keywords:** China-Africa health aid, Health diplomacy, Global health, Development assistance, Foreign aid

## Abstract

**Background:**

China has played an increasing role in development aid across Africa. Most recently, China has increased its external investments through the Belt and Road Initiative, China’s signature infrastructure and trade drive to link China to Asia and Africa. This is likely to result in continuing growth of China’s investment in health in sub Saharan Africa. While conflicting opinions have been raised regarding the motivation and value of these investments, few data have been solicited from those directly involved in China-Africa health aid. We conducted a qualitative study to collect information on perceptions and opinions regarding Chinese-supported health related activities in Africa through in-depth interviews among local African and Chinese participants in Malawi and Tanzania.

**Results:**

Our findings reveal shared experiences and views related to challenges in communication; cultural perspectives and historical context; divergence between political and business agendas; organization of aid implementation; management and leadership; and sustainability. Participants were broadly supportive and highly valued Chinese health aid. However, they also shared common insights that relate to challenging coordination between China and recipient countries; impediments to communication between health teams; and limited understanding of priorities and expectations. Further, they share perspectives about the need for shaping the assistance based on needs assessments as well as the importance of rigorous reporting, and monitoring and evaluation systems.

**Summary:**

Our findings suggest that China faces similar challenges to those experienced by other longstanding development aid and global health donors. As it continues to expand cooperation across Africa and other regions, it will be important for China to consider the issues identified through our study to help inform collaborative and effective global health assistance programs. The insights garnered from this research are not only relevant to China’s engagement in Africa but for other global health assistance donors as well.

## Background

Health aid has been an important and long-term component of China’s engagement with Africa. It has grown substantially and reflects China’s efforts to improve the accessibility of health services, strengthen public health systems, and build the capacity of health workers in African countries [1, 2]. However, with its increasing role as a major global health donor, China also confronts similar challenges faced by other actors in global health funding, including the prioritization of resources; coordination of health investments (donor preferences versus recipient priorities); financial and programmatic accountability; shortage of health workforce; limited strength of national governance in recipient countries, and the ever-present challenge of sustainability of these investments [3–7]. Some have described China’s support to Africa as opportunistic, perceiving China’s engagement in foreign relationships as a mechanism for economic or political gain [7–9]. China’s governance is described as a “soft power”; a noncoercive strategy in which culture, political ideology, economic strength, and foreign policy are used to persuade other nation-states to adopt aligning values [10–12]. Others view China’s motivation as altruistic; however, China’s “no strings” approach to aid has raised some concern that it may unintentionally enable human rights violations [10, 12, 13].

In response to these challenges, in 2018 China pursued efforts to reform its foreign aid structure with the establishment of the China International Development Cooperation Agency (CIDCA) [7]. CIDCA aims to reduce fragmentation between ministries and other agencies previously involved in aid, aiming to enhance oversight and accountability in implementation, improve coordination between governmental bodies, and to reduce tensions between diplomacy and economic and commercial ventures [14]. This new agency also oversees the Belt and Road Initiative (BRI), China’s signature infrastructure and trade enterprise that links China to Asia and Africa through new ports and overland trading routes. This initiative also seeks to promote international cooperation and offer potential opportunities for health investment, capacity building, knowledge sharing of health threats, and approaches to enhance health governance [15].

However, many questions remain regarding the scope of Chinese aid, its effectiveness, and the global governance principles that guide the conceptualization and implementation of such efforts. In response, we conducted a qualitative study to identify opportunities and challenges with regards to China’s foreign aid; specifically, a deeper understanding of how it prioritizes and allocates health aid, how this aid fits within the specific African country’s health systems and needs, and how to assess the effectiveness of Chinese foreign aid.

## Methods

### Study design

This was an exploratory qualitative study to gain understanding about China’s health related activities in Malawi and Tanzania. In-depth interviews were conducted to seek insights from individuals who work directly and/or are familiar with the dynamics and processes of the health aid delivery in these countries. Using a semi structured interview guide, the questions aimed to capture their experiences with various types of China-Africa health activities, perceptions of successes and challenges, as well as to identify lessons learned and suggestions for shaping and enhancing the programs to increase their potential impact. A total of 58 interviews were conducted in Dar es Salaam and Zanzibar, Tanzania and Lilongwe, Malawi between May and September of 2016.

### Study participants

A combination of purposive and snowball sampling methods was used to recruit participants in Malawi and Tanzania. Participants included both Chinese nationals who were working in Malawi and Tanzania and local Malawian and Tanzanian representatives of various sectors. Participants were selected based on their (1) direct engagement with China sponsored health projects and/or awareness but not direct engagement with such projects; (2) age must be 18 years or older; and (3) fluency in either English or Mandarin.

Overall participant characteristics are shown in Tables 1 and 2. Interviews were conducted with 29 participants in Malawi (20 Malawians and 9 Chinese) and 29 participants in Tanzania (13 Tanzanians and 16 Chinese). The health sector represented the largest percentage of total participants, about 58%, of which 36% were clinical staff (physicians, nurses, other healthcare workers); about 20% health facility administrative staff (project director, administrators, health facility managers); and 2% staff of disease specific programs. The government sector, representing health and social welfare ministries and Chinese embassy staff, totaled approximately 34%. Participants from non-governmental organizations (NGOs) (3.4%) and research/academic institutions (5.1%) accounted for the smallest percentages.

**Table 1:** Participant Characteristics by Sector

SECTOR	MALAWI		TANZANIA		
	LOCAL	CHINESE	LOCAL	CHINESE	
Health	**9**	**8**	**3**	**14**	**34 (58.6%)**
Government (ministry, embassy)	**11**	**1**	**6**	**2**	**20 (34.4%)**
Academic/research			**2**		**2 (3.4%)**
NGO			**2**		**2 (3.4%)**
**TOTAL PARTICIPANTS**	**20 (34.4%)**	**9 (15.5%)**	**13 (22.4%)**	**16 (27.5%)**

**Table 2:** Participant Characteristics by Role or Function

	LOCAL	CHINESE	LOCAL	CHINESE	
Health team – physician, nurse, other medical staff		**8**		**13**	**21 (36.2%)**
Health facility staff – project director, manager, administrative	**9**		**3**		**12 (20.6%)**
Ministry or embassy representative	**11**	**1**	**6**	**2**	**20 (34.4%)**
NGO representative			**2**		**2 (3.4%)**
Other (disease specific program, research center)			**2**	**1**	**3 (5.1%)**

### Data collection

Semi-structured interview guides were developed in English for Malawian and Tanzanian participants and a Mandarin interview guide for Chinese participants. Both guides included introduction questions followed by modular questions. In the English guide, there were modular questions for (1) individuals who are directly engaged with China-sponsored projects, i.e. health facility staff, disease specific program and (2) individuals who are aware but not directly engaged with such projects, i.e. academic/research, government ministry, non-governmental organizations (NGO). The Mandarin guide included modular questions for (1) individuals from the Chinese embassies in Malawi and Tanzania and (2) Chinese medical/health teams.

Interviews were facilitated by two researchers, both fluent in English and Mandarin; audio recorded; translated and/or and transcribed into English. Translations and transcriptions were done by approved third party services that specialize in health and medical fields.

### Ethical considerations

Ethical approval for interviews was given by Columbia University Irving Medical Center Institutional Review Board and local ethics committees in Malawi and Tanzania. All participants agreed to a written consent in the presence of a witness prior to the interview. Participants consented to have interviews audio recorded.

### Data analysis

Transcripts were analyzed using inductive content analysis to identify shared notions by the participants [16]. The research team met to review and discuss preliminary findings from interviewer memos, interview guides used, and previous literature reviews. Thereafter, transcription and translation of participant’s interviews were uploaded to ATLAS.ti, a computer program for qualitative analysis. A preliminary subsample of several randomly selected transcripts was reviewed closely to obtain an initial understanding of the data. From this subsample, researchers considered major topics, high frequency wording, and repeated terms [17]. Researchers evaluated, matched, and categorized the data generally using memos and open coding. Preliminary codes were identified and defined. These preliminary findings were used to construct a raw coding scheme for further analysis [18].

All interviews were analyzed in ATLAS.ti line by line using an inductive process to organize data. The coding scheme from the content of the preliminary subsample was expanded on, developed, and organized into a series of categories, themes, and codes to accurately represent the participants’ perspectives and practices. Transcripts were reviewed by morphology, context, semantic expression, diction, and syntax in addition to content. Themes were based on participant’s intent, similar implied and expressed meaning, congruent references, and inference to larger ideas. Further, the relationships and interactions between themes were evaluated to enhance significance. Additional codes were identified and defined as necessary. Codes were used to expand and define key themes which fell into one of six categories. This structure (Table 3) was used to improve understanding and generate knowledge [19, 20].

**Table 3:** Themes and Sub-themes of Content Analysis

**Theme**	**Subtheme**	**Codes**
**Communication**	strained working relationships	barriers to aid, attitudes of Chinese workers, language barrier, culture, training, politics
	language barriers	language barrier
	varying social norms and mores	culture, value
	patronization of African HCW	attitudes of Chinese workers, aid person, burnout
**Cultural Perspectives and Historical Context**	lack of cultural relativity	culture, value, attitudes of Chinese health workers
	power dynamics	awareness, colonial history, corporate, politics
	lack of historical context	barriers to aid, future, colonial history, politics
	absence of trust	barriers to aid, attitudes of Chinese workers, trust, not observed, impact
**Divergence between Political and Business Agendas**	opportunities for private investment	corporate, politics, value
	moral hazard and adverse selection	politics, impact, trust, economic development, corporate, value
	unequal distribution of political capital (i.e. reference how Africans feel obliged to accept donations)	colonial history, politics, economic development, corporate, impact, value
	concealed motivation	impact, attitudes of Chinese workers, future
	influence of multilateral organizations	politics, lack of external collaboration, colonial history, trust, value
**Organization of Aid Implementation**	countries’ policies and procedures misaligned	barriers to aid, procedures, coordination failure, capacity failure
	substandard or expired donations	idle resources, material resources, not observed, substandard, logistical failure, barriers to aid, lack of regulation
	country needs not met	in-country needs, material resources, infrastructure, training, recommendations, economic development
	inadequate training	training, sustainability, material resources, aid person, barriers, politics, country needs
**Management and Leadership**	poor country and donor collaboration	logistical failure, barriers to aid, lack of external collaboration
	inadequate consultation	barriers to aid, consultation
	lack of central authority	barriers to aid, capacity failure, logistical failure, sufficient internal communication
**Sustainability**	wasted, lost, or destroyed resources	idle resources, language barriers, training, material resources, inadequate consultation, sufficient internal communication, lack of external collaboration, not observed
	lack of M&E protocol	evaluation, sustainability
	aid worker burnout	sustainability, evaluation, burnout, attitudes of Chinese health workers

## Results

Overall, our study findings demonstrated  African participants’ deep appreciation and regard for China’s aid and acknowledged the importance of China-Africa cooperation in Malawi and Tanzania. However, all participants also offered insight to the challenges and opportunities within the aid paradigm. Study data revealed key themes relating to China’s health aid that fit into six major categories: (1) communication, (2) cultural perspectives and historical context, (3) organization of aid implementation, (4) management and leadership, (5) divergence between business and political agendas, and (6) sustainability. The categories, themes, and supporting coding scheme were examined to identify relationships and crossover as well as to cluster similar or repetitive themes.

### Communication

While Chinese and African healthcare workers expressed mutual professional respect, language barriers challenged engagement. Participants described both verbal and nonverbal miscommunication, such as mannerisms, gestures, and etiquettes unique to each culture that are distinctive and could be misinterpreted. When verbal and nonverbal communications were strained, relationships between African and Chinese participants were described as stressful. Healthcare workers, both Chinese and African, expressed that difficulty in communicating with each other hampered successful aid delivery.

Due to this complex interplay, some participants felt the struggles to communicate effectively perpetuated a power differential with Chinese workers having more status due to their professional clout. African healthcare workers felt that the misunderstandings that arose from communication issues were interpreted as a judgmental, prejudice, and possibly patronizing attitude by Chinese healthcare workers. Chinese health workers had a different perspective; they felt that their lack of institutional training and support to prepare them for working in African contexts created immense stress, hindering their effectiveness and making healthy communication between them and their African coworkers less of a priority.

“*language is an obstacle, including cultural concepts, … including some etiquette, customs, … Our gesture(s) and way of expression may not be easy for [Africans] to understand.” – Chinese Participant*

### Cultural perspectives and historical context

Individual barriers to communication stemmed from systemic misunderstandings that have existed between the involved parties for some time. African participants reported that Chinese counterparts needed to improve cultural relativity as without it, communication challenges can arise. Within cultural perspectives, participants discussed the importance of historical context, specifically Africa’s colonial history and the replicated power dynamics that emanate from the individual level to a higher systemic level. Participants felt it was important to consider the historical relationship and that the risk of not doing so resulted in the replication of previous (inequitable) colonial relations. African participants noted that China’s current aid delivery appeared consistent with the approaches taken by previous colonial powers. Participants described China’s power as having more political capital, economic resources, institutional development, and educational attainment. This inequity in institutional power limited the capacity for trust and further strained the interpersonal relationships needed for successful programmatic implementation.

“*You [China] just came here to build, just making money here, has not brought benefits to the locals. This is why they took Africa as a new colony.” – African Participant*

### Divergence between political and business agendas

In relation to institutional trust, African participants perceived corporate influence in China’s health aid, i.e. interests of Chinese private corporations and State-Owned Enterprises (SOEs) in expanding their markets, as a key driver of China’s interest in Africa. African participants noted this imbalance in political capital and the influence and authority China has over the recipient states. African participants described their experience of this imbalance and cited examples, such as their inability to deny donations or aid, even if they felt that the specific aid offered was not needed.

Additionally, African participants perceived China as having hidden motivations centered on expansion of its political sphere of influence in low-and middle-income countries and among global health stakeholders. Some believed that China’s foreign aid was an attempt to gain more political power. However, others recognized that a set of external forces including multi-lateral organizations have a substantial impact on politics and aid implementation, often driving health initiatives and influencing aid priorities.

“*If you want to change—if you want this aid, you have to make sure you include this in your law … aid with strings attached … . Politically, you have to … receive it with a smile. Yeah. Even if it’s a plastic smile.” - African Participant*

### Organization of aid implementation

African participants indicated that the process of acquiring and delivering aid is sometimes unclear and inconsistent and that these processes do not always map directly to in-country systems and needs. Participants noted that this disjuncture often led to delivery of donations that were considered substandard and beyond expiration dates resulting in wasted resources.

China’s limited consultation to identify country needs and goals compounded with the misaligned local policies and procedures perpetuated barriers to aid. Prior to delivery, China was described as rarely consulting the recipient country to assess their needs. Participants felt that these oversights left other crucial needs in recipient countries unaddressed.. This meant that the donations delivered were often surplus, unusable, or did not meet local regulatory requirements. China’s aid often came with no notice and limited communication. This created confusion among recipient state’s agencies, as well as adding administrative burdens to process the aid received.

African participants repeatedly recommended more training opportunities for healthcare workers. Training was often cited as a mechanism to encourage autonomy and sustainability. Participants cited the value of the trainings provided by Chinese health workers and their desire to have more training in other areas, especially in health administration. Training was thought to be a potentially effective mechanism to improve and sustain health outcomes through “train the trainer” programs and through specific training on how to use donated equipment. The absence of training technicians when donating medical equipment was a recurring barrier to effective utilization of such equipment.

“ *… it'd be great if there is a clear responsibility … (an) organizational structure. (For) China … as an international donor … it will be almost impossible to clearly understand all the internal organizational structures in (a country) … it's also difficult to find the right person or right division to talk to … (unless) there is a clear responsibility and framework established internally in government.” – African Participant*

### Management and leadership

Challenges in leadership and management exacerbate issues in coordination, consultation, and delivery of China’s aid. Participants suggested more systematic and managerial support from China. Gaining institutional and economic support beyond donations and services from China would improve African countries structure, organization, management, and leadership.

Additionally, Chinese foreign aid was described as strictly bilateral with China reluctant to collaborate with other donors. Participants felt this approach created duplicated services and goods, gaps in need, and new challenges for recipient countries. However, African participants noted that  recipient countries also face their own struggles with management and leadership in coordination of the aid they receive, necessitating the need for streamlined responsibility and strong governance.

“*I see a gap in (Chinese) understanding (our) system and (our) situation. So I think … If we could find a way that they learn a little bit … . They should be told how .. the system work(s), what is the policy, what are the regulations, when it comes to the health organization - how the hospital is organized, how do we get material, what are the challenges, and possible challenges in working … .*” – *African Participant*

### Sustainability

A major theme that emanated from the participant interviews was the limited attention to sustainability as a result of challenges in communication, management and leadership, and organization of implementation. China’s aid agenda recognizes the importance of sustainability, however, study participants described the need to streamline inputs, activities, and participation. This revealed why sustainability is challenging.

Participants described various experiences of wasted resources and effort, from a lack of streamlined activities to communication gaps. Some noted product instructions were not interpreted in the recipient’s language; training on the use of products was not provided; and replacement parts for highly technical equipment were often missing. This in turn placed undue financial, administrative, and logistical burdens on the recipient countries to resolve these oversights.

Participants were encumbered with how best to utilize donations that were not always requested or appropriate to real needs and reported having to expend considerable staff time, money, and resources to do so. The limited consistency and dependability of China’s aid and its sometimes mismatch to recipient country priorities and policies limited the potential for sustainability. Participants noted that many of their concerns regarding aid delivery could have been addressed with prior consultation, assessment, or policy alignment as well as the need for putting in place monitoring and evaluation mechanisms. Finally, Chinese health workers highlighted their mandated foreign service, lack of institutional support, and inconsistent resources as reasons for burnout. This had led to low professional motivation and morale, high turnover rates, and thus reduced potential for sustainability.

*“ … looking ahead in the long-term [needs], for sustainability. So [China should be] taking into consideration the approach of working more broadly, working through a task force or a committee, to be more inclusive. Ensuring that there is transparency … to get better buy-in, a longer-term plan which everybody [China and recipient country] can buy into, so the plans have action available to all stakeholder.”- African Participant*

## Discussion

### Complexity of culture and communication

Overall, participants expressed respect for their respective African and Chinese counterparts and believed that they could learn from one another. However, they cited the need to improve communication strategies to overcome the linguistic and cultural barriers. Poor cultural exchanges and communication in international partnerships increase the difficulty of global governance and effective assistance [21, 22]. In this study, several healthcare workers expressed difficulty engaging and connecting with their counterparts. These communication strains could be attributed to the need for cultural relativity to enhance working professional relationships which would secondarily improve employee motivation, commitment, productivity, well-being, and safety [22], especially in international settings [23]. However, from the Chinese participants’ perspective, relationship strain was attributed to their workload and lack of institutional support for their individual and professional needs. The struggles of Chinese healthcare workers and its impact on foreign aid have been previously reported [6, 24]. Healthcare worker burnout is common in foreign aid [25] and could explain the stress between staff, as it has in other international aid settings [26].

These individual communication challenges were also reflected at the system level. Multilateral engagement between China and other global health donors could reduce donor duplication, minimize gaps in resources, and improve sustainability. This echoes existing recommendations for improving sustainability and governance in international settings [27, 28]. In addition, the need for institutional communication and engagement would help streamline coordination of aid from specific donors. Prior to the establishment of CIDCA, China had a complex governance structure comprised of more than twenty central line ministries, commissions, and agencies all of which had significant responsibilities in the delivery of aid, and many operated independent of one another. This led to complicated management and heightened miscommunication resulting in oversight, coordination issues, and systematic challenges [29]. Some have described China’s foreign aid as lacking transparency, accountability, and goodwill [30]. Adhering to these principles can strengthen working relationships, maximize efficiency, establish mutual respect, and foster equality [27, 31, 32]. Leaders can play an important role in transforming the nature of communications between donors and recipient countries by strengthening communication channels at the institutional level to help defuse conflicts among different units of operation [28].

### Limited transparency fosters political distrust and suspicion

China’s approach to foreign aid has been described as low risk because it combines aid with commercially oriented trade and investment [33–35]. This risk averse approach to foreign aid in an effort to preserve self-interests is not uncommon [36]. There is sizable literature examining China’s motivation for providing foreign aid [37, 38] with some indicating that China’s motivation for delivering aid was not anchored in goodwill, but rather focused on enhancing private, corporate, or State-Owned Enterprise investments, its economic gains, and advancing its international reputation [34, 39]. The consequences of this risk averse approach can be detrimental to the recipient country [40].

This concern about China’s motivation with regards to its foreign aid has been raised in reports from several countries, including Cambodia [41, 42], Ethiopia [43], and other African countries [44]. China’s reduced consultation and collaboration, limited attention to African countries’ management and fiscal concerns, and tendency toward regional favoritism have been noted as challenges to effective aid [45]. China’s secondary economic and political priorities that motivate aid allocation handicap their aid efficiency [37]. This style of governance unfortunately limits trust, cooperation, collaboration, and communication, all important characteristics of impactful aid [46, 47].

### Limited coordination between China and recipient countries hinders the impact of the health aid

The importance of collaboration and coordination between China and recipient African countries was repeatedly cited by participants. This limitation resulted in misaligned policies and priorities. When this happens, valuable products are lost and the recipient country bears the burden of additional personnel time, opportunity cost, and financial constraints to resolve the miscoordination [28]. Additionally, recipient African states struggle internally with logistics and coordination [35]. This reiterates the need for donors to coordinate strategies and priorities to improve aid management systems [48]. China should reframe its foreign aid to offer organizational and managerial support to recipient states in order to streamline coordination, enhance communication, and improve effectiveness. Overall, simplifying and harmonizing governmental policies and procedures for receiving foreign aid along with transparency and accountability systems could improve existing aid frameworks [48, 49].

### China’s misaligned health aid has consequences that can perpetuate a culture of dependence

The misaligned policies and procedures, miscommunication, and lack of consultation waste human and material resources. As a result, recipient countries assume the administrative burden of needing to account for the redundancy, policy incoherence, and inefficient use of resources [29, 50, 51]. Our study findings suggest that China’s aid delivery may not be sustainable, if it fails to establish procedures of transfer to the recipient state and reduces their ability to establish autonomy and control over time [52, 53]. Further, without concerted efforts to do so, it will only exacerbate the lack of central authority that exists in some African countries [14, 35]. International development and response should identify consistent leadership and resources and engage with local stakeholders if sustainability is to be achieved [14].

### Opportunities and recommendations

The identified challenges in China’s aid to Africa noted in this study continue to impede the effectiveness of Chinese foreign aid, even after CIDCA’s establishment [6, 7]. However, there are several political and diplomatic opportunities for improving the potential value of this aid. Specifically, it behooves China to recognize issues of power, politics, and interest groups with regards to its current aid paradigm [27]. First, there is the need for insuring that its own national strategy for foreign aid is better incorporated into government guidelines on health diplomacy. This would improve CIDCA’s capacity as well as aid overall by aligning internal resources, goals, and strategies [2, 6]. Second, CIDCA should expand its scope to include the need for the development of relationships with other donors in order to improve multilateral collaborations [6, 7]. Third, implementation strategies should be tailored to the development plans of recipient countries and should align with the sustainability goals of other donors [7]. This requires CIDCA to evolve into a top-down institutional system of aid that can ensure policy alignment; create and improve communication platforms; and define goals [6]. Additionally, China should utilize the various forums it supports such as the South-South Cooperation to engage recipient countries in an equitable discourse on global health governance [54]. Fourth, to improve transparency, China should work on defining protocols for data collection and analysis on foreign aid as well as for implementing rigorous needs assessments and monitoring and evaluation mechanisms [6, 7]. Calls for transparency, oversight, and defined criteria within Belt and Road Initiatives reiterate this priority [10, 55]. Finally, China should consider restructuring their strategy for deploying and supporting healthcare workers abroad. Specifically, China should create a system that supports their providers working abroad and create support systems to address their emotional needs and sense of isolation; concerns about career advancement; and ensure consistent and uniform working and living environments [6].

Other opportunities exist for China to improve both interpersonal and system communication. For example, its aid frameworks should adapt to utilize local individuals, organizations, and culture; encourage communities to participate in delivery [28]; encourage recipient countries to engage in the discussion and planning of aid delivery; and promote recipient countries’ autonomy over aid projects [14, 45]. It has been noted that cross-cultural exchanges in international aid settings can encourage human cultural diversity, mutual respect, and consultation which promotes good governance in public affairs [56, 57]. Additionally, efforts should incorporate mechanisms of cultural humility, a practicable skill that acknowledges the diversities of groups, emphasizes mutual respect, and considers the inevitable power dynamics to facilitate an attitude of acceptance and collaboration between healthcare workers [23, 58, 59]. However, communication challenges can become more complex within systems of bureaucracy, management, and leadership [14]. Opportunities to improve system communication include sharing ownership of the aid process between donors and recipients, monitoring and evaluation processes, alignment of systems and priorities through consultation, implementation of accountability measures, and harmonized costs between donors’ and recipients’ systems [58, 60].

### Limitations

This study has several limitations. Firstly, the data were collected in 2016 before the establishment of CIDCA and may not be fully representative of the current situation. Secondly, the research interviewers were Chinese, and this may have influenced the responses received from participants. Thirdly, the cross-cultural nature of the study design and its utilization of translators may have introduced barriers to interpreting and conveying participant experiences fully.

## Conclusions

As China continues to expand cooperation with African countries, it faces many of the same challenges experienced by other major donors in global health aid including coordination, prioritization, recipient country engagement, as well as cross-cultural communication challenges. However, China is in a unique position to meet these challenges by applying and building on lessons learned from its own and others’ missteps and increasing its engagement with key stakeholders, including other donors and recipient countries. Streamlining coordination, management, consultation, and logistics are clear priorities that are recognized in the mandate of the new CIDCA. Now is the time to take this mandate to action.

## Data Availability

The transcripts and coding schemes that support the findings of this study are available on request from the corresponding author WES. The data may be available upon request for partnering research or projects but is not available due to data protection laws.

## Abbreviations

BRI: Belt and road initiative
CIDCA: China international development cooperation agency
IDI: In-depth interview
NGO: Non-governmental organization
SOE: State-owned enterprise
UNICEF: United Nations children’s fund
WHO: World health organization
